# The Effects of Partially or Completely Substituted Dietary Zinc Sulfate by Lower Levels of Zinc Methionine on Growth Performance, Apparent Total Tract Digestibility, Immune Function, and Visceral Indices in Weaned Piglets

**DOI:** 10.3390/ani9050236

**Published:** 2019-05-13

**Authors:** Yuhuai Xie, Qing Zhang, Lixue Wang, Yuxi Wang, Zhenfeng Cheng, Zaibin Yang, Weiren Yang

**Affiliations:** 1 Shandong Provincial Key Laboratory of Animal Biotechnology and Disease Control and Prevention, College of Animal Science and Veterinary Medicine, Shandong Agricultural University, Tai’an 271018, Shandong, China; yuhuai12@163.com (Y.X.); qizhang@sdau.edu.cn (Q.Z.); lxwang1119@163.com (L.W.); zfcheng@sdau.edu.cn (Z.C.); yangzb@sdau.edu.cn (Z.Y.); 2 Lethbridge Research Center, Agriculture and Agri-Food Canada, Lethbridge, AB T1J 4B1, Canada; Yuxi.Wang@AGR.GC.CA

**Keywords:** zinc sulfate, zinc methionine, apparent total tract digestibility, serum metabolites, immune functions, weaned piglets

## Abstract

**Simple Summary:**

This study was conducted to assess the effects of five diets with different doses and sources of zinc (Zn) on the growth performance (average daily gain, average daily food intake and gain to feed ratio), apparent total tract digestibility of nutrients, serum metabolites and immune functions of weaned piglets. The control diet contained 100 mg/kg inorganic Zn from ZnSO_4_. The total dose of Zn in experimental diets was lower than that of the control diet, and the inorganic Zn from ZnSO_4_ was gradually replaced by organic Zn from ZnMet. Therefore, the experimental diets were a basal diet containing 75 + 12.5, 50 + 25, 25 + 37.5, and 0 + 50 mg/kg Zn from ZnSO_4_ and ZnMet, respectively. No differences were observed in growth performance, nutrient digestibility and serum metabolites. However, Zinc digestibility and parameters relating to body immune functions were improved when at least 50 mg of inorganic Zn was replaced by organic Zn. Thus supplementing 50 mg of inorganic Zn from ZnSO_4_ plus 25 mg of organic Zn from ZnMet to piglets would be the best strategy to benefit the immune system and maintain growth performance under the conditions of the current study.

**Abstract:**

The study aimed to evaluate the effects of replacing zinc sulfate (ZnSO_4_) with a lower level of zinc methionine (ZnMet) on the growth performance, apparent total tract digestibility (ATTD) of nutrients, serum metabolites and immune functions of weaned piglets. Thirty-five weaned Duroc × Landrace × Large White male piglets (10.69 ± 0.26 kg) were randomly allotted to five diets. The control diet was supplemented with 100 mg/kg of Zn from ZnSO_4_, and experimental diets included 75 + 12.5, 50 + 25, 25 + 37.5, and 0 + 50 mg/kg of Zn from ZnSO_4_ and ZnMet, respectively. The results showed that no differences were observed in growth performance, ATTD of nutrients and serum metabolites among treatments, while serum white blood cell count, lymphocyte count, IgM contents and spleen index were higher (*p* < 0.01) in piglets fed with 50 + 25 mg/kg of Zn. Zinc digestibility (*p* < 0.05), IgA content (*p* < 0.001) and thymus index (*p* < 0.05) were increased when at least 50% of ZnSO_4_ was replaced by ZnMet. All the results indicated that using a lower level of ZnMet in weaned piglet’s diet instead of ZnSO_4_ had no adverse impacts on ATTD of nutrients and serum metabolites; and a 50 + 25 mg/kg of Zn (from ZnSO_4_ and ZnMet, respectively) diet showed the best advantages for parameters relating to immune functions.

## 1. Introduction

Zinc (Zn), one of the essential mineral elements, is an indispensable component of transcription factors and numerous metalloenzymes in the metabolic processes of organisms [1], particularly these processes involved in carbohydrate, protein and lipid metabolism [2]. Additionally, Zn plays an important role in the development of both innate and adaptive immune systems [3]. According to the nutrient requirement of swine (NRC 2012), the Zn requirement of weaned piglets decreases from 100 to 60 mg/kg diet when pig’s body weight (BW) increases from 5 to 50 kg. However, due to the growth factors effect or the large safety margins, more than 10 to 20 times of inorganic Zn, such as zinc oxide (ZnO) or zinc sulfate (ZnSO_4_) was commonly applied in the diet to maximize the performance of piglets after weaning [4]. The long-term supplementation with high doses of inorganic Zn may impact the growth performance in young pigs due to the interaction with other minerals [5]. Furthermore, a high concentration of Zn in combination with antibiotics appears to accelerate microbial antibiotic resistance in the microbiota community in piglet intestines [6,7].

It has been reported that the excessive usage of inorganic mineral Zn may lead to the low bioavailability of other minerals such as copper, which, in turn, has negative effects on the metabolism of iron [8]. In addition, due to the low bioavailability of the inorganic Zn sources caused by the bonding of Zn with fiber or phytates in basal diets [9], excessive Zn supplementation results in more Zn excretion, which will have negative effects on the environment [10]. Although ZnSO_4_ is used as a typical inorganic source, dietary zinc supplementation of weaned piglets can be achieved by different sources. Organic Zn sources are forms of minerals that bond to amino acids/peptide/protein, which could reduce the complexation between phytic acid and Zn [11]. The previous studies have demonstrated that the organic forms of Zn have potential higher bioavailability compared with the inorganic sources in diets containing phytate and fiber, which means organic Zn can provide alternative pathways for high absorption and low mineral excretion [12]. Therefore, the enhanced bioavailability of organic Zn sources can reduce the total supplementation of Zn in piglets’ diets and, in turn, can alleviate environmental heavy metal contamination of some regions.

The objectives of the current study were to evaluate the effects of reducing total dietary Zn content (below 100 mg/kg) by using lower levels of ZnMet instead of ZnSO_4_ on growth performance, apparent nutrient digestibility, hematological parameters, serum metabolites, and some parameters relating to immune functions of weaned piglets.

## 2. Material and Methods

### 2.1. Animal, Dietary Treatment and Experimental Design

The experimental protocols for animals were consistent with the Guidelines for the Care and Use of Laboratory Animals and approved by the Ethical Commission (Approval Number: S20180058) of Shandong Agricultural University Animal Nutrition Research Institute.

Thirty five 30-d-old crossbred (Duroc × Landrace × Large White) male piglets weaned at 28-d old with 10.69 ± 0.26 (mean ± SD) kg of BW were used in the 42-d experiment. Piglets were randomly allocated into five groups and assigned to five dietary treatments with 7 piglets per treatment. There was no difference in initial average body weight between treatments. The corn-soybean meal-based basal diet, analyzed to contain 46.28 mg/kg of Zn, was formulated to meet or exceed all nutritional requirements for weaned piglets (10–35 kg, NRC 2012), except for Zn. The composition of the basal diet is listed in Table 1. The control diet was obtained by supplementing the basal diet with 100 mg Zn/kg as ZnSO_4_. The 100 mg Zn/kg from ZnSO_4_ in the control diet was gradually replaced by Zn from ZnMet at the rate of 2:1 (Zn from ZnSO_4_: Zn from ZnMet) to obtain the other four experimental diets, which yielded the following four concentrations of supplemented Zn: 75 + 12.5, 50 + 25, 25 + 37.5 and 0 + 50 mg/kg diet (mg Zn from ZnSO_4_ + mg Zn from ZnMet). The feed-grade ZnMet (zinc content is 17.20%) and ZnSO_4_·H_2_O (zinc content is 35.02%) were obtained from Shandong Longxin Feed Co., LTD. (China).

The experiment was arranged as a completely randomized design. Piglets were fed individually in metabolism cages (1.55 m × 0.85 m) in a temperature controlled house at the farm of Shandong Agricultural University (Tai’an, China). The initial room temperature was set at 29 °C and gradually decreased at the rate of 1.5 °C/week to 24 °C, which was maintained until the end of the experiment. The metabolism cage was constructed from stainless steel and equipped with a stainless steel nipple waterer and feeder. Animal management was the same during the whole experimental periods. The experiment consisted of a 7-d adaptation period and a 42-d data collection period. The diets in the adaptation period were gradually replaced by the experimental diets. Animals were fed daily for ad libitum intake and had free access to water throughout the whole experiment period.

Piglets were weighed individually after fasting overnight on d 0 (37-d-old) and d 42 (79-d-old) during the experiment to calculate the average daily gain (ADG). Feed intakes, orts and spillage were recorded every day to determine the average daily feed intake (ADFI). Feed efficiency was calculated by the gain to feed ratio (G/F). Each diet was sampled weekly and composited to three samples for chemical analysis.

### 2.2. Determination of Apparent Total Tract Digestibility

Feces were collected without losses from each piglet during d 22- d 28 using the method described by Fouhse et al. (2017) [13]. The feces collected each time were mixed thoroughly, weighed, mixed with 10% H_2_SO_4_ at a ratio of 10:1 (w/v) and stored at −20 °C. The 7-d collected feces were composited for each piglet. Approximately 100 g feces were subsampled after thorough mixing, and dried at 65 °C for 48 h for chemical analysis. Feed offered and orts of each piglet were weighed and recorded daily for calculation of feed intake. Diet was also subsampled daily, composited for each group and dried at 65 °C for 48 h for chemical analysis.

All diets and feces samples were ground to pass a 1.00 mm screen prior to laboratory analysis. Gross energy (GE) content was determined using the Parr adiabatic bomb calorimeter (Model 6200, Parr Instruments Co, Moline, IL, USA) described by Zhang et al. (2012) [14]. Dry matter (DM), crude protein (CP), and organic matter (OM) were analyzed according to the procedures described by Wang et al. (2011) [15]. Analysis of Zn in diets and feces was performed by flame atomic absorption spectrophotometry (SpectrAA 220, Mulgrave, Victoria, Australia). All methods were based on AOAC [16]. Apparent total tract digestibility (ATTD) of DM, OM, CP, GE and Zn was calculated with the following formula [17]:

$$\mathrm{ATTD}\;\left(\%\right)=\frac{\mathrm{DMI}\times \mathrm{NCD}-\mathrm{FW}\times \mathrm{NCF}}{\mathrm{DMI}\times \mathrm{NCD}}\times 100$$

where DMI is the dry matter intake (kg), NCD is the nutrient (DM, OM, CP, GE or Zn) content diet (DM basis), FW is the output of feces per day (kg), and NCF is the nutrient (DM, OM, CP, GE or Zn) content of the feces (DM basis).

### 2.3. Determinations of Blood Metabolites and Immunoglobulin

On the first and last day of the experiment, blood samples were collected from each piglet after fasting for 8 h. Approximately 10 mL of blood sample was taken from the left jugular vein into a vacutainer. For the analysis of hematological indices, about 1.0 mL of collected whole blood was transferred to 5-mL vacuum tubes (Becton Dickinson, Franklin Lakes, NJ, USA) treated with tripotassium ethylenediaminetetraacetic acid (K_3_EDTA) anticoagulant. The tubes were immediately placed in an ice box and analysis was completed within 4 h after sampling. The samples were analyzed using a hematology analyzer (Sysmex KX-21, Sysmex Corporation, Japan) for the counts of white blood cell (WBC), lymphocyte (LYM), intermediate cell (MID) and granulocyte cell (GRA) using procedures described by Weiss et al. (2011) [18].

The remaining blood samples were transferred into a coagulation-promoting tube (BD, New York, USA), kept at room temperature for 1.0 h and centrifuged for 15 min (1000× *g*, 4 °C) to obtain serum. The serum was subsequently stored at −20 °C for the analysis of serum metabolites and immunoglobulin.

Total protein (TP), albumin (ALB), blood urea nitrogen (BUN), alkaline phosphatase (ALP), triglyceride (TG), and lactate dehydrogenase (LDH) in the prepared serum were determined by an automatic biochemistry blood analyzer (Olympus AU5400, Tokyo, Japan) using procedures recommended by the manufacturer. The content of different immunoglobulin subsets including immunoglobulin A (IgA), immunoglobulin G (IgG), and immunoglobulin M (IgM) was determined by immunoturbidimetry method using corresponding commercial kits (Jiancheng Biochemical Reagent Co., Nanjing, China) according to the manufacturer’s instructions.

### 2.4. Determinations of Visceral Indices

All piglets were humanely euthanized by intracardiac injection of sodium pentobarbital (50 mg/kg of BW) [19] at the end of the feeding experiment. The organs including liver, pancreas, thymus, and spleen were removed immediately and weighed. The visceral indices were then calculated using the following formula:

Visceral index (g/kg) = Organ weight (g)/Live body weight (kg).

### 2.5. Data Calculations and Statistical Analysis

All data were presented as means ± SEM. Data were analyzed by ANOVA using the general linear models procedure of SAS 9.2 (SAS Inst. Inc., Cary, NC, USA) with treatment as the fixed effect and individual piglet as the experimental unit. The effect of the total Zn supplementation was determined by linear and quadratic effects. The significance of differences among treatments was tested using Duncan’s multiple range tests by least square means with the PDIFF procedure of SAS. Difference was declared to be statistically significant when *p* < 0.05. Tendency was declared with *p*-values between 0.05 and 0.10.

## 3. Results

### 3.1. Growth Performance and ATTD of DM, OM, CP, GE and Zn

Animal growth performance data are presented in Table 2. Briefly, piglets supplemented with different levels or sources of Zn had a similar ADG and G/F (*p* > 0.05). However, ADFI tended to be decreased with increasing levels of ZnMet (Linear, *p* = 0.084) and tended to reach a minimun value at 50 + 25 mg/kg Zn (Quadratic, *p* = 0.098). Similarly, as shown in Table 3, the average ATTD of DM, OM, CP and GE across the treatments was 86.43 ± 0.78, 88.69 ± 0.69, 80.92 ± 0.48 and 83.03 ± 0.81% respectively. Replacing Zn from ZnSO_4_ with reduced amount of Zn from ZnMet had no effect (*p* > 0.05) on ATTD of DM, OM or GE regardless the amount being replaced. However, the ATTD of CP tended to decrease linearly (*p* = 0.061) with the increase of ZnMet substitution levels. In addition, the ATTD of Zn was significantly increased (*p* < 0.05) in piglets fed diets supplemented with 50 + 25, 25 + 37.5 and 0 + 50 mg/kg of Zn compared with the control group, and showed linear (*p* < 0.01) and quadratic (*p* < 0.01) effects with increasing levels of ZnMet.

### 3.2. Serum Metabolites, Immune Hematological Indices and Immunoglobulins

Irrespective of the treatments, all piglets had similar TP, ALB, ALP, TG, and LDH contents in the serum (*p* > 0.05), as present in Table 4. However, the serum BUN content increased up to 25 + 37.5 mg/kg Zn, but decreased with 0 + 50 mg/kg Zn (Quadratic, *p* < 0.05). In addition, both total WBC and LYM count peaked at 50 + 25 mg/kg Zn (Quadratic, *p* < 0.05). As the levels of substitution increased, the total GRA count tended to decrease linearly (*p* = 0.078), although no difference was observed (*p* > 0.05) in the count of MID among treatment groups.

As shown in Table 5, with increasing substitution levels of ZnMet, the serum IgA concentration showed a linear increase (*p* < 0.001) and peaked at 25 + 37.5 mg/kg Zn (Quadratic, *p* < 0.001). However, the IgM concentration increased and reached the maximum value at 50 + 25 mg/kg Zn (Quadratic, *p* < 0.01). All groups of piglets had a similar serum concentration of IgG (*p* > 0.05).

### 3.3. Visceral Indices

The visceral indices are shown in Table 6. The results demonstrated that the thymus index increased linearly (*p* < 0.05) up to 50 + 25 mg/kg Zn, then tended to level off (Quadratic, *p* = 0.088) with increasing levels of ZnMet. In addition, the spleen relative weight was heavier (*p* < 0.01) in piglets fed diet supplemented with 50 + 25 mg/kg of Zn than for other groups of piglets. However, the relative weights of liver and pancreas were not affected by the treatments (*p* > 0.05).

## 4. Discussion

Zn is an essential dietary nutrient for swine, and it is considered as a critical element in maintaining the structure of metalloproteins such as growth hormone and insulin [1]. The native Zn in common feedstuffs is poorly available to pigs because of its complexation with phytate or fiber, and therefore the diets must be supplemented with Zn in the swine industry. However, the piglet’s rations are often formulated to contain Zn in higher concentrations than recommended doses to promote growth and reduce diarrhea, which causes the excess zinc to be released to the environment.

In the present study, the effects of using low levels of ZnMet instead of ZnSO_4_ on growth performance, apparent nutrient digestibility, and parameters relating to immune functions of weaned piglets were assessed. Our observation showed that the similar ADG and feed efficiency were obtained when the 100 mg/kg Zn as ZnSO_4_ was gradually replaced with lower levels of Zn as ZnMet. This result indicated that the supplementation of 50 mg/kg of organic Zn from ZnMet to the basal diet would be sufficient to meet the requirement to sustain the normal growth of piglets from weaning until 49 d post-weaning, although the total supplementation of Zn content decreased from 100 to 50 mg/kg. Our results showed a similar response compared with the limited available research. Van et al. (2003) reported that there was no difference on growth performance in weanling pigs supplemented with 80 mg/kg of organic Zn (ZnMet or Zn lysine) compared to the pigs supplemented with 160 mg/kg of inorganic Zn from ZnSO_4_, which was similar to our study [20]. Wang et al. (2010) found that weanling piglets supplemented with 100 mg/kg Zn from zinc glycine chelate or 3000 mg/kg Zn as ZnO could achieve similar growth performance [21]. In the study of Case et al. (2002), nursery pigs fed 500 mg/kg Zn from Zn-amino acid complexes grew faster than pigs fed 500 mg/kg inorganic Zn, but there was no difference compared with pigs fed 3000 mg/kg inorganic Zn [4]. These similar results are likely due to the enhanced bioavailability of organic Zn [22,23]. Moreover, the similar DM, OM and GE digestibility of piglets observed in this study demonstrated that complete replacement of 100 mg/kg inorganic Zn from ZnSO_4_ with 50 mg/kg organic Zn from ZnMet had no adverse impact on the nutrient digestibility. Zn deficiency primarily influences protein metabolism in fast-growing pigs [24]. Interestingly, the ATTD of CP tended to be decreased linearly as the substitution levels increased. In addition, the Zn digestibility was also enhanced when at least 50 mg/kg inorganic Zn was replaced by ZnMet. It has been reported that increased apparent absorption and the retention of organic zinc sources were due to the different absorption pathways of different forms of Zn [22]. ZnMet can be absorbed in methionine absorption pathways and deposited together with methionine into the proteins of tissues [25], and this may be the reason for the enhancement of Zn digestibility in our results. Thus, although the supplemented total levels of zinc were decreased, the similar ATTD of nutrients were still observed in the present study.

To some extent, the concentration of serum TP and ALB can reflect the metabolic status of hepatic protein in response to dietary changes in weaned piglets. Serum BUN is the main product of protein metabolism and indicates the whole status of amino acid metabolism and utilization in body [26]. This study demonstrated that partial or complete replacement of the inorganic Zn source (ZnSO_4_) by ZnMet did not affect the serum TP and ALB content, which was generally in agreement with previous studies in weaned piglets [27], cows [28], and rats [29]. However, the quadratic effect of organic Zn substitution on serum BUN concentration was observed as the organic Zn ratio increased. Serum BUN content increased as the organic Zn ratio increased, peaked at 25 + 37.5 mg/kg Zn and then decreased as the organic Zn ratio further increased. These results might be due to the fact that Zinc is an important factor of enzymes involved in protein metabolism [30], and the increase of the organic Zn ratio might be beneficial to the improvement of enzyme activity when the total Zn level decreased. Total glycerol is an important energy source, as most tissues derive energy by metabolizing the TG synthesized in liver [27,31]. The serum ALP and LDH content are associated with the health status of hepatic and nephritic cells [32]. No differences were observed on serum TG, ALP and LDH contents, and all these parameters were within the normal range of healthy piglets. This again suggested that replacing inorganic Zn with low levels of organic Zn could be sufficient to maintain the normal nutrient metabolism and health status of organs, although the levels of total Zn content were lower than those recommended by NRC. Similar to our results, the addition of low levels (20 or 10 ppm) of Zn from Zn glycinate did not affect the serum TG, ALB and ALP concentration in broilers compared with the treatment supplemented with 40 mg/kg Zn from ZnSO_4_ [33]. Therefore, these results indicated that using a low level of ZnMet instead of ZnSO_4_ had no adverse effects on the synthesis of protein and fat metabolism in piglets in the present study. 

Dietary requirement for trace minerals for optimizing immune function may be higher than for the growth requirements [20], yet limited study in piglets was reported regarding the low levels of Zn sources combinations on immunity. This study evaluated the immune functions from several parameters to determine the effects of low levels of combinations of different Zn sources on immunity. It is well known that WBC and LYM are important parts of the immune system. White blood cells fight infections that cause phagocytosis and deliver pathogens to phagosomes, where the pathogens are eliminated [34]. Lymphocytes play an important role in regulating the immune system through the proliferation and differentiation of B cells and T cells [35]. Prasad et al. (1996) reported that Zn deficiency has an adverse effect on the proliferation and maturity of lymphocytes [36]. Zn deficiency can affect the function of neutrophils, monocytes and macrophages [37]. In the present study, all the values of hematological indices (total WBC, LYM, MID, and GRA) were within the normal ranges for piglets. Both the counts of WBC and LYM showed quadratic effect as the inclusion levels of ZnMet increased, and both peaked at 50 + 25 mg/kg Zn. Although, the GRA count showed a linear decrease trend, yet no quadratic effect was observed. These results suggested that 50 mg Zn as ZnSO_4_ plus 25 mg Zn as ZnMet/kg diet was the optimal combination for the proliferation of immune cells. Leite et al. (2018) reported that altering zinc source from ZnSO_4_ to zinc amino acid complex increased the number of T cells in non-infected intestinal tissue, which was in agreement with our results [38]. All these observations on the improvement of immune cells in these studies might be due to the high digestibility and bioavailability of ZnMet [39], although the total Zn content was decreased.

Immunoglobulins including IgA, IgG, and IgM, play an important role in immune system. Increasing immunoglobulins content can improve the function of clearing pathogens in immune reaction [40]. In this study, the linear and quadratic effects for serum IgA content and the quadratic effect for serum IgM content were observed with the increase of organic Zn ratio. The treatments supplemented with 50 + 25 and 25 + 37.5 mg/kg Zn seemed to be the optimal Zn sources combination for the improvement of immunoglobulins. Consistent with these results, Moghaddam et al. (2009) reported that dietary supplementation of ZnMet instead of inorganic zinc source improved the immunity through increasing IgM and IgG content [41]. The spleen and thymus, as immune organs, play crucial roles in the immune function of the body. The spleen is the site of lymphocyte growth, division and differentiation, and it initiates the immune reaction to blood-borne antigens [42]. The thymus regulates the immune function by cytokines and thymus hormone secreted by stromal cells [43]. It has been demonstrated that the weight of the spleen and thymus can be enhanced by immune system activators [44], and the organ indices will change in response to the organism’s nonspecific immunity [45]. As evidenced in study herein, the relative weight of thymus increased linearly up to 50 + 25 mg/kg Zn, then tended to level off. The spleen index was also improved when piglets were supplemented with 50 +25 mg/kg Zn. These results were in good agreement with a previous report which demonstrated that thymus weight was increased when dietary Zn from ZnO was replaced by 75% or 100% ZnMet in broiler chicks [41]. This is probably because of the higher absorption capacity of ZnMet that allows lower inclusion rates of Zn and makes it easer for Zn or methionine to participate in immune regulation. Overall, these results suggested that organic Zn source could significantly improve the parameters relating to body immune function of weaned piglets by replacing inorganic Zn source in diet. 

## 5. Conclusions

In conclusion, decreasing supplemented dietary inorganic Zn source (100 mg/kg) from ZnSO_4_ by gradually replacing the Zn source with a lower dose of Zn from ZnMet had no negative effects on growth performance, nutrient digestibility and common serum metabolites of piglets from weaning to 49 d post-weaning. Moreover, Zn digestibility, blood and organ parameters related to the body immune function of the piglets were enhanced when at least 50 mg/kg Zn from ZnSO_4_ was replaced with Zn from ZnMet. Thus, in the current study, supplementing 50 mg/kg of inorganic Zn from ZnSO_4_ plus 25 mg/kg of organic Zn from ZnMet to piglets fed corn-soybean meal diet is the best strategy to benefit the animal’s immune system and maintain growth performance.

## Figures and Tables

**Table 1:** Composition and nutrient contents of basal diet.

Item	Content
Ingredient, %	
Corn	63.00
Soybean meal	24.00
Soybean oil	1.00
Corn gluten meal	3.00
Wheat flour	5.00
Calcium monophosphate	1.35
Limestone	0.79
Sodium chloride	0.48
Compound enzyme preparation	0.03
L-Lys HCl	0.35
Vitamin-trace mineral premix ^1^	1.00
Total	100.00
Analyzed composition	
DE ^2^, MJ/kg	13.90
CP ^2^, %	19.09
Ca, %	0.70
Total P, %	0.60
Lys, %	1.35
Met, %	0.46
Met + Cys, %	0.77
Thr, %	0.88
Zn, mg/kg	46.28

^1^ Supplied per kilogram of complete diet: 11,000 IU vitamin A, 2300 IU vitamin D3, 80 IU vitamin E, 4.4 mg vitamin K3, 4.4 mg thiamine, 11 mg riboflavin, 34 mg d-pantothenic acid, 59.5 mg niacin, 330 mg choline, 0.9 mg folic acid, 0.5 mg biotin, 55 µg vitamin B12, 40 mg vitamin B5, 4.0 mg vitamin B6, 40 mg Mn as manganese sulfate, 130 mg Fe as ferrous sulfate, 15 mg Cu as copper sulfate, 0.40 mg I as potassium iodide, 0.3 mg Se as sodium selenite. ^2^ DE, digestible energy; CP, crude protein.

**Table 2:** Effects of dietary substitution of zinc sulfate (ZnSO_4_) by low levels of zinc methionine (ZnMet) on growth performance of 37- to -79-d-old piglets ^1^.

Item	Control	ZnSO_4_ + ZnMet, mg/kg				SEM	*p*-Value	*p*-Value	
	100 + 0	75 + 12.5	50+25	25 + 37.5	0 + 50			Linear	Quadratic
ADG, g	538	541	495	512	519	11	0.706	0.195	0.214
ADFI, g	1222	1219	1114	1129	1158	24	0.478	0.084	0.098
G/F ^2^	0.44	0.45	0.44	0.46	0.45	0.011	0.992	0.727	0.933

^1^ Values are means and pooled SEMs, *n* = 7. ^2^ ADG, average daily gain; ADFI, average daily food intake; G/F, gain to feed ratio; SEM, standard error of mean.

**Table 3:** Effects of dietary substitution of ZnSO_4_ by low levels of ZnMet on apparent total tract digestibility (ATTD) of nutrients for 37- to -79-d-old piglets ^1^.

Item	Control	ZnSO_4_ + ZnMet, mg/kg				SEM	*p*-Value	*p*-Value	
	100 + 0	75 + 12.5	50 + 25	25 + 37.5	0 + 50			Linear	Quadratic
DM, %	86.59	86.40	87.79	84.34	87.05	0.775	0.977	0.745	0.926
OM, %	88.45	88.23	89.46	88.24	89.07	0.692	0.973	0.586	0.860
CP, %	81.71	81.77	82.03	79.09	80.01	0.412	0.172	0.061	0.174
GE, % ^2^	82.00	83.13	83.99	82.13	83.90	0.812	0.897	0.439	0.710
Zn, %	29.12 ^b^	32.76 ^ab^	34.11 ^a^	35.25 ^a^	35.52 ^a^	0.592	0.037	0.002	0.004

^a, b^ Means within a row with different superscripts differ significantly (*p* < 0.05).^1^ Values are means and pooled SEMs, *n* = 7. ^2^ DM, dry matter; OM, organic matter; CP, crude protein; GE, gross energy.

**Table 4:** Effects of dietary substitution of ZnSO_4_ by low levels of ZnMet on serum metabolites and hematological indices of 37- to -79-d-old piglets ^1.^

Item	Control	ZnSO_4_+ZnMet, mg/kg				SEM	*p*-Value	*p*-Value	
	100 + 0	75 + 12.5	50 + 25	25 + 37.5	0 + 50			Linear	Quadratic
TP, g/L	56.04	57.02	57.52	55.74	60.20	0.755	0.533	0.129	0.224
ALB, g/L	35.95	36.44	34.62	34.82	36.42	0.549	0.661	0.838	0.545
BUN, mmol/L	3.28	3.52	3.72	3.86	3.54	0.123	0.748	0.120	0.042
ALP, IU/L	82.17	82.00	81.83	80.33	84.20	2.117	0.986	0.811	0.830
TG, mmol/L	0.26	0.21	0.25	0.21	0.19	0.019	0.712	0.135	0.337
LDH, IU/L	563	570	577	577	588	18.214	0.587	0.171	0.407
WBC, 10^9^/L	6.36 ^b^	6.78 ^ab^	7.54 ^a^	6.88 ^ab^	6.76 ^ab^	0.122	0.041	0.433	0.044
LYM, 10^9^/L	2.29 ^b^	2.46 ^b^	3.05 ^a^	2.24 ^b^	2.07 ^b^	0.078	0.021	0.423	0.045
MID, 10^9^/L	1.32	1.36	1.28	1.32	1.30	0.072	0.997	0.992	0.992
GRA, 10^9^/L ^2^	3.76	3.64	3.84	3.56	3.44	0.062	0.190	0.078	0.121

^a, b^ Means within a row with different superscripts differ significantly (*p* < 0.05). ^1^ Values are means and pooled SEMs, *n* = 7. ^2^ TP, total protein; ALB, albumin; BUN, blood urea nitrogen; ALP, alkaline phosphatase; TG, triglyceride; LDH, lactic dehydrogenase; WBC, white blood cell; LYM, lymphocyte; MID, intermediate cell; GRA, granulocyte.

**Table 5:** Effects of dietary substitution of ZnSO_4_ by low levels of ZnMet on IgA, IgM and IgG of 37- to -79-d-old piglets ^1^.

Item	Control	ZnSO_4_+ZnMet, mg/kg				SEM	*p*-Value	*p*-Value	
	100 + 0	75 + 12.5	50 + 25	25 + 37.5	0 + 50			Linear	Quadratic
IgA	0.510^b^	0.521^b^	0.572 ^a^	0.604 ^a^	0.589 ^a^	0.007	<0.001	<0.001	<0.001
IgM	0.483^b^	0.493 ^b^	0.518 ^a^	0.496 ^ab^	0.480 ^b^	0.003	0.012	0.936	<0.01
IgG	4.005	4.890	4.305	4.380	4.435	0.147	0.459	0.742	0.693

^a, b^ Means within a row with different superscripts differ significantly (*p* < 0.05). ^1^ Values are means and pooled SEMs, *n* = 7.

**Table 6:** Effects of dietary substitution of ZnSO_4_ by low levels of ZnMet on visceral indices of 37- to -79-d-old piglets ^1^.

Item	Control	ZnSO_4_+ZnMet, mg/kg				SEM	*p*-Value	*p*-Value	
	100 + 0	75 + 12.5	50 + 25	25 + 37.5	0 + 50			Linear	Quadratic
Thymus	0.283 ^b^	0.300 ^b^	0.357 ^a^	0.307 ^ab^	0.350 ^a^	0.007	0.026	0.034	0.088
Spleen	1.883 ^b^	1.807 ^b^	2.577 ^a^	1.850 ^b^	1.813 ^b^	0.057	<0.01	0.889	0.195
Liver	25.09	23.70	24.04	24.66	23.36	0.572	0.869	0.507	0.805
Pancreas ^2^	1.843	2.063	2.003	1.707	1.940	0.084	0.694	0.781	0.922

^a, b^ Means within a row with different superscripts differ significantly (*p* < 0.05). ^1^ Values are means and pooled SEMs, *n* = 7. ^2^ Visceral index (g/kg) = Organ weight (g) /Live body weight (kg).
